# *Mycobacterium neoaurum* Contamination

**DOI:** 10.3201/eid1108.040861

**Published:** 2005-08

**Authors:** Xiang Y. Han

**Affiliations:** * University of Texas, Houston, Texas, USA

**Keywords:** Rapidly progressive dementia, Mycobacterium neoaurum, Central nervous system, Meningitis, Rheumatoid arthritis

**To the Editor:** In reviewing "Rapidly Progressive Dementia due to *Mycobacterium neoaurum* Meningoencephalitis," by Heckman et al. (*1*), I found, contrary to the authors' conclusion, that *M. neoaurum* was more likely a contaminant than a cause. First, within the granulomatous brain lesions, the strongest evidence for the authors' conclusion, no acid-fast bacilli were isolated or identified on special stains; thus, the Kochs postulates were not satisfied. Rather, the lesions were likely rheumatoid nodules. Longstanding rheumatoid arthritis commonly causes granulomalike rheumatoid nodules. I did a PubMed search using "rheumatoid nodule in the brain" and 7 articles were found (*2**,**3*). A "rheumatoid endarteritis" search found 25 articles. Heckman et al. failed to exclude or discuss this possibility.

Second, *M. neoaurum* is a rare environmental mycobacterium that grows in ≤2 days on sheep blood agar and is not difficult to culture. As the authors stated, there have been 8 reports of this organism, 7 isolated from blood and 1 from urine. The blood isolates were associated with either central venous catheter or intravenous drug use. Thus, *M. neoaurum* is of low virulence and unlikely to cause spontaneous infection in tissue unless inoculated accidentally, perhaps. Third, polymerase chain reaction (PCR) is exquisitely sensitive and prone to contamination. The problem is worse when bacterial DNA is amplified by using highly conserved primers. The PCR reagents, from the Taq polymerase (of bacterial origin) to water, contain sufficient, despite minute quantity, bacterial DNA to be amplified (*4*). Although direct sequencing of the amplicon is often blurry because of its low quantity and mixed content, when cloned, each amplicon may be ligated to the vector and proliferates and gets sequenced later.

Therefore, I believe the presence of *M. neoaurum* DNA, not the organism itself, represented contamination. Generally, drawing cause-disease conclusion based on PCR sequencing needs vigilance to satisfy the modified Koch postulates (*5*).
