# Supplementary material for: Mycobacterium neoaurum Contamination
Source: Emerg Infect Dis. 2005 Aug;11(8):1316–7. doi: 10.3201/eid1108.040861 (PMC3320498; doi:10.3201/eid1108.040861)
Supplement: Related texts

# Reply: *Mycobacterium neoaurum* Contamination

**DOI:** 10.3201/eid1108.050630

**Authors:** George A. Heckman, Cynthia Hawkins, Andrew Morris, Lori L. Burrows, Catherine Bergeron

**Affiliations:** * Chedoke Campus and Freeport Health Centre, Kitchener, Ontario, Canada;; † University of Toronto, Toronto, Ontario, Canada;; ‡ McMaster University, Hamilton, Ontario, Canada;; § Toronto Western Hospital, Toronto, Ontario, Canada

**In Response:** In response to our report on a case of rapidly progressive dementia (1,2), Dr. Han argues that *Mycobacterium neoaurum* was "more likely a contaminant than the cause" and that the actual cause of death was most likely rheumatoid pachymeningitis. Dr. Han bases his argument on the absence of positive acid-fast stains or mycobacterial cultures and his assessments that the identification of *M. neoaurum* DNA was due to contamination and that the pathologic findings represented rheumatoid nodules.

The inability to stain or culture an organism in this case is not unusual, as paucibacillary mycobacterial infections, such as tuberculous lymphadenitis and leprosy, are common (3,4). Though the possibility is not inconceivable, environmental contamination is unlikely, because tissue samples were positive with *M. neoaurum*–specific primers, whereas controls containing identical reagents but no tissue were not.

Dr. Han expresses a valid concern that rheumatoid pachymeningitis was not given due consideration. Rheumatoid pachymeningitis is a rare complication of rheumatoid arthritis, in which patients may exhibit headache, cranial neuropathies, focal deficits, seizures, or cognitive dysfunction (5,6). Rheumatoid pachymeningitis usually, but not exclusively, occurs in patients with long-standing rheumatoid arthritis characterized by erosive disease and extra-articular manifestations, although the systemic disease may be quiescent when neurologic complications arise. Cerebrospinal fluid analysis is generally nonspecific. Magnetic resonance imaging may show prominent meningeal enhancement. Pathologic features may include vasculitis, rheumatoid nodules, and meningeal inflammation, with the latter 2 features being most common (5). The dura may demonstrate inflammation with fibrinoid necrosis (6). We reviewed the pathologic specimens of this case and confirmed the presence of abundant giant cells, endarteritis proliferans, and, most notably, extensive caseation necrosis typical of mycobacterial infection. We found no evidence of rheumatoid nodules, dural inflammation, or fibrinoid necrosis.

Though this case does not satisfy Kochs postulates, neither do most novel infectious diseases. Substantial international efforts were required to satisfy the postulates in the case of SARS (7). In this case, the identification of DNA from a "rare environmental mycobacterium" in a patient with overwhelming pathologic evidence of mycobacterial infection provides strong, though not foolproof, evidence of a possible causal role.

## References

[1] Han XY. *Mycobacterium neoaurum* contamination. Emerg Infect Dis. 2005;11:1316–7.1611058210.3201/eid1108.040861PMC3320498

[2] Heckman GA, Hawkins C, Morris A, Burrows LL, Bergeron C. Rapidly progressive dementia due to *Mycobacterium neoaurum* meningoencephalitis. Emerg Infect Dis. 2004;10:924–7.1520083310.3201/eid1005.030711PMC3323241

[3] Chao SS, Loh KS, Tan KK, Chong SM. Tuberculous and nontuberculous cervical lymphadenitis: a clinical review. Otolaryngol Head Neck Surg. 2002;126:176–9. 10.1067/mhn.2002.12191211870349

[4] Ustianowski AP, Lockwood DNJ. Leprosy: current diagnostic and treatment approaches. Curr Opin Infect Dis. 2003;16:421–7. 10.1097/00001432-200310000-0000814501994

[5] Kato T, Hoshi K, Sekijima Y, Matsuda M, Hashimoto T, Otani M, Rheumatoid meningitis: an autopsy report and review of the literature. Clin Rheumatol. 2003;22:475–80. 10.1007/s10067-003-0788-014677033

[6] Tan HJ, Raymond AA, Phadke PP, Rozman Z. Rheumatoid pachymeningitis. Singapore Med J. 2004;45:337–9.15221051

[7] Osterhaus ADME, Fouchier RAM, Kuiken T. The aetiology of SARS: Koch's postulates fulfilled. Philos Trans R Soc Lond B Biol Sci. 2004;359:1081–2. 10.1098/rstb.2004.148915306393PMC1693394
